# Leveraging anatomy to improve safety and efficacy in temple augmentations—a case series of 20 patients

**DOI:** 10.3389/fsurg.2025.1603177

**Published:** 2025-09-25

**Authors:** Bruna S. F. Bravo, Leonardo Gonçalves Bravo, Bárbara Fouraux Gouvea, Gabriel L. T. Alves

**Affiliations:** Department of Dermatology, Bravo Private Clinic, Rio de Janeiro, Brazil

**Keywords:** temple augmentation, soft tissue fillers, facial anatomy, hyaluronic acid, facial rejuvenation

## Abstract

**Introduction:**

Temple augmentation has become a cornerstone in full-face aesthetic rejuvenation, yet traditional techniques often face limitations, including aesthetic limitations and the risk of vascular complications. This study explores a novel interfascial injection technique, aiming to improve both the safety and efficacy of soft tissue fillers in the temporal region by leveraging anatomical knowledge.

**Materials and methods:**

A total of *n* = 20 female patients, aged 26–75, received injections using Ultra Volume Rennova®, a high-viscoelastic dermal filler. The filler was administered with a 22G × 5 cm cannula into the interfascial plane, located between the superficial and deep temporal fascia. Ultrasound imaging was used to confirm accurate filler placement. The access point for injections was the infrazygomatic arch, targeting the anterior third of the temporal region.

**Results:**

Favorable outcomes in terms of volumization and lifting effects in the upper third of the face were reported in all patients. Fifteen patients reported mild pain post-injection, and two reported moderate pain, with no serious adverse events or hematomas observed.

**Conclusion:**

This interfascial injection technique offers a safer and more effective alternative to traditional subcutaneous or supraperiosteal methods. By leveraging the anatomical relationship between the zygomatic arch and the deep temporal fascia, the procedure reduces the risk of vascular complications while achieving natural and lasting aesthetic results. Further studies are warranted to validate these findings and assess long-term outcomes.

## Highlights

- A novel interfascial injection technique was developed for the temporal region, enhancing safety by reducing the risk of vascular complications while delivering natural volumization and lifting effects.
- In a study involving 20 patients, no serious adverse events occurred, with minimal pain reported, underscoring the technique's safety and efficacy.
- This method optimizes filler placement by utilizing the continuity between the zygomatic arch periosteum and deep temporal fascia, allowing safe access via the zygomatic arch. It offers a superior alternative to subcutaneous or supraperiosteal injections, potentially improving outcomes in facial rejuvenation procedures.

## Introduction

Facial rejuvenation through minimally invasive procedures has gained popularity due to its ability to deliver natural-looking results with minimal downtime. The temporal region – initially often neglected by both patients and aesthetic providers – receives an increasing amount of attention as its importance in the overall aesthetic perception is being recognized (1, 2). Traditional methods of filler injections in the temporal area including subcutaneous and supraperiosteal injections often faced challenges in achieving optimal outcomes due to safety concerns and anatomical limitations. Specifically, the risk of vascular complications, such as vision loss following arterial injections, necessitated a reevaluation of the injection techniques (3).

In this study, we present a novel technique that leverages regional anatomical principles to target the interfascial plane—a potential space of loose areolar connective tissue between the superficial and deep temporal fasciae. This approach aims to enhance treatment efficacy in the temporal region while improving safety by minimizing the risk of injury to critical vascular structures, such as the superficial temporal artery, thereby providing aesthetic practitioners with a method grounded in anatomical precision.

## Materials and methods

This prospective study included a cohort of 20 female patients, aged between 26 and 75 years, who underwent temple augmentation with an advanced high-viscoelastic dermal filler. This study was conducted in a private clinical setting as part of routine facial rejuvenation procedures and internal quality assurance. All patients provided written informed consent for both the treatment and the anonymized use of their clinical data, including photographic and ultrasonographic documentation, for scientific publication. No investigational product was used; all treatments involved a commercially available hyaluronic acid filler designed for deep tissue integration and structural support (Ultra Volume Rennova, Rennova®, Goiânia, Brazil). According to national regulations in Brazil and prevailing ethical standards for private clinical practice, retrospective analysis of anonymized data collected for quality monitoring and educational purposes does not require formal IRB approval. The study was conducted in accordance with the ethical principles outlined in the Declaration of Helsinki.

The filler was delivered using a 22G × 5 cm blunt-tip cannula (Rennova®, Goiânia, Brazil), allowing for atraumatic distribution of the product within the targeted anatomical compartment. All injections were meticulously administered within the interfascial plane, anatomically defined as the space between the superficial temporal fascia and the deep temporal fascia, which is composed of loose areolar tissue. Although the superficial and deep temporal fasciae fuse at the superior zygomatic arch, they separate immediately superior to the arch, creating an interfascial compartment of loose connective tissue (4). This plane is of particular clinical interest due to its capacity for accommodating volumetric augmentation while minimizing the risk of vascular compromise or irregular contouring. A 22G 50 mm cannula was inserted at the entry point on the superior border of the zygomatic arch between the medial and its central third on the longitudinal way. The cannula was inserted at the entry point at an angle of 90 degrees; initially the tip of the cannula touched the periosteum of the zygomatic arch and only then was directed towards the temporal region. After entering the temple in the interfascial layer, the cannula was guided towards the temporal crest and then retracted to the central region of the temple where a bolus injection of around 0.2 ml of hyaluronic acid was made.

**Figure 1:**
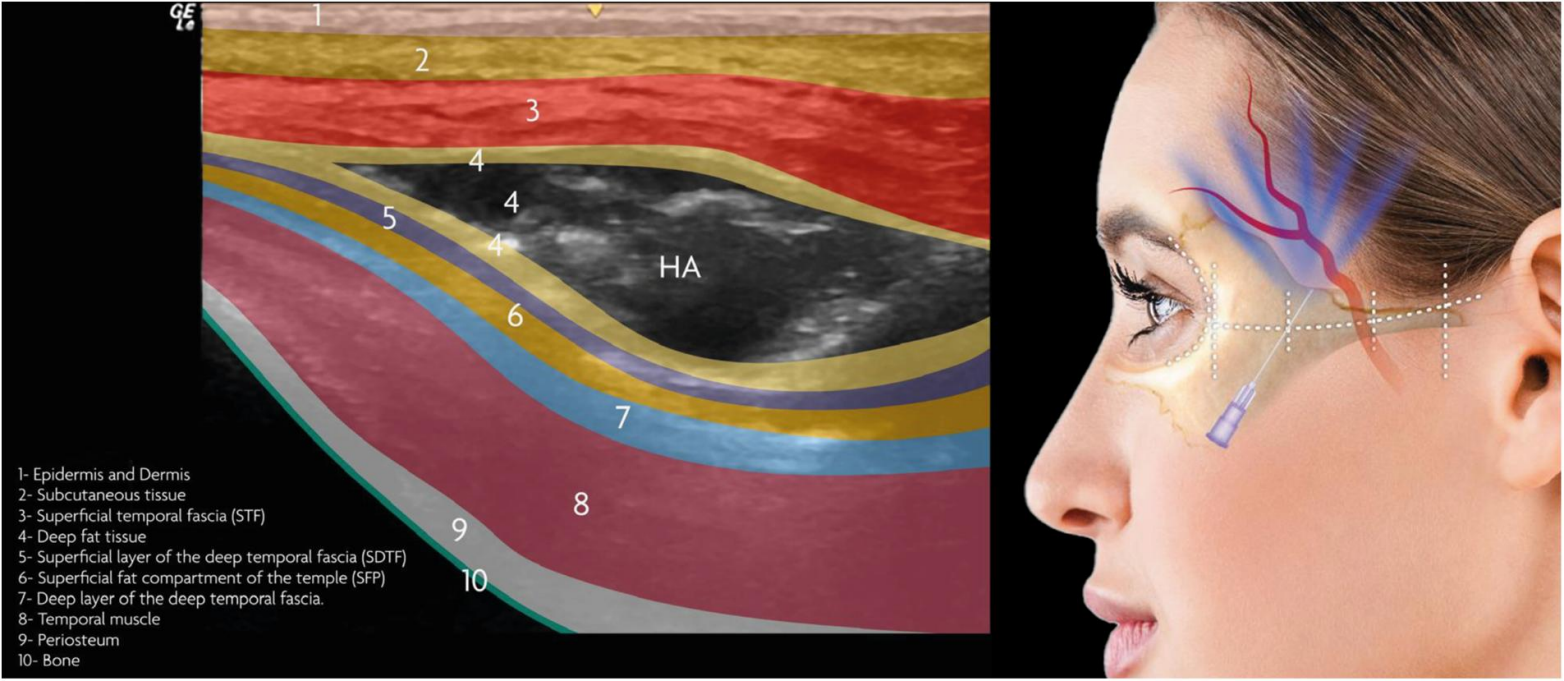
Illustration depicting skin layers with HA applied in the interfascial layer, alongside the injection technique showing the access point at the anterior third of the zygomatic arch.

**Figure 2:**
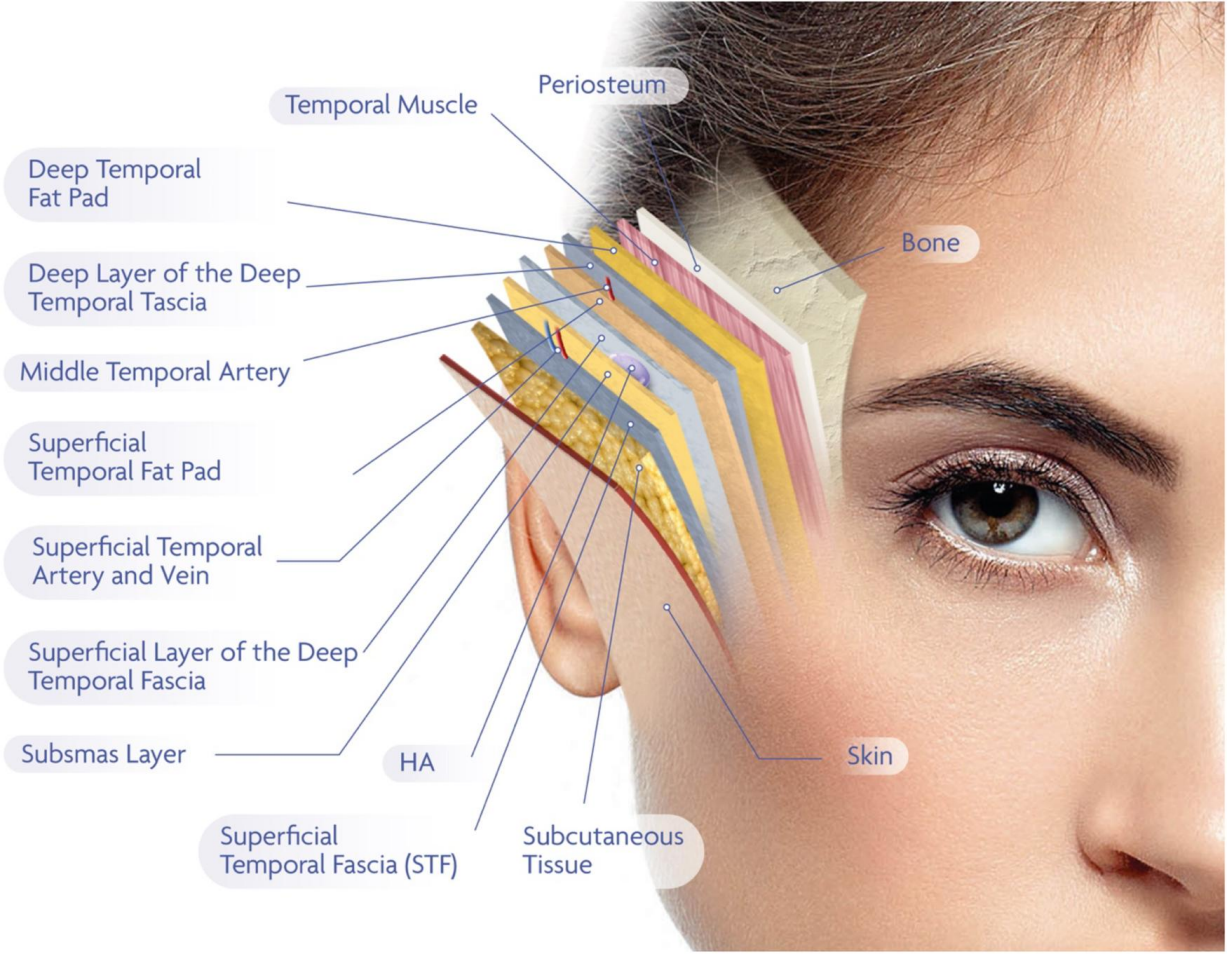
This figure presents a layered anatomical schematic of the temporal region, relevant to interfascial filler injections. From superficial to deep, the structures are arranged as follows: the skin and subcutaneous tissue overlie the superficial temporal fascia, also referred to as the temporoparietal fascia. Beneath this lies the SubSMAS layer, a loose areolar connective tissue plane. This is followed by the superficial layer of the deep temporal fascia, which envelops the superficial temporal fat pad. The middle temporal artery, and in some cases the middle temporal vein, course through or adjacent to this compartment. Below this, the deep layer of the deep temporal fascia encloses the deep temporal fat pad and covers the underlying temporalis muscle, which itself lies directly above the periosteum and temporal bone. The interfascial plane—targeted in this study for filler deposition—is defined as the space between the superficial and deep laminae of the deep temporal fascia. This compartment is composed of loose connective tissue and offers an anatomically favorable location for hyaluronic acid (HA) placement. It provides sufficient depth to minimize the risk of visible product irregularities while maintaining a safe distance from major vascular structures. The terminology used in this illustration reflects the most current anatomical literature, while acknowledging existing terminological variability among anatomical schools. The schematic is intended to offer both conceptual clarity and clinical relevance for aesthetic practitioner.

To ensure accuracy and reproducibility, real-time ultrasonographic imaging was employed to confirm the precise localization of the filler within the interfascial space. High-resolution ultrasound visualization was performed in accordance with standardized imaging protocols, allowing for verification of the injection plane, assessment of filler distribution, and exclusion of inadvertent vascular compromise. More specifically, using an 18 MHz “hockey stick” transducer (GE L8-18i-SC 8–18 MHz California-USA) coupled to the GE Logic Ultrasound (NextGen, California-USA, 2023), mapping was performed. The patient was placed on a stretcher in the supine position. The temporal region was cleaned using sterile gauze and a 3% aqueous chlorhexidine solution. (Riohex, Rioquimica, Brasil). The 8–18 Hz hockey stick probe was placed longitudinally parallel and right above to the cannula starting from the entry point at the zygomatic arch to the temporal crest. The lubricant gel used for the examination was a sterile gel (Sterile Aquasonic Ultrasonic gel, PARKER, Brazil), to avoid contamination of the entry site and the video recording was made with the probe stationary in the same longitudinal direction. The transducer was placed in B-mode initially to map all anatomical structures, followed by power Doppler mode to visualize vascular structures and confirm a safe injection corridor. Finally, the location and planes were marked in mm according to its distance from the supraperiosteal plane at a 90-degree angle and differentiated between them. Real-time ultrasonography was used to verify that the filler appeared as an anechoic layer between the two hyper-echoic fascial planes (Figures 1, 2 and Supplementary Video S1).

## Results

**Figure 3:**
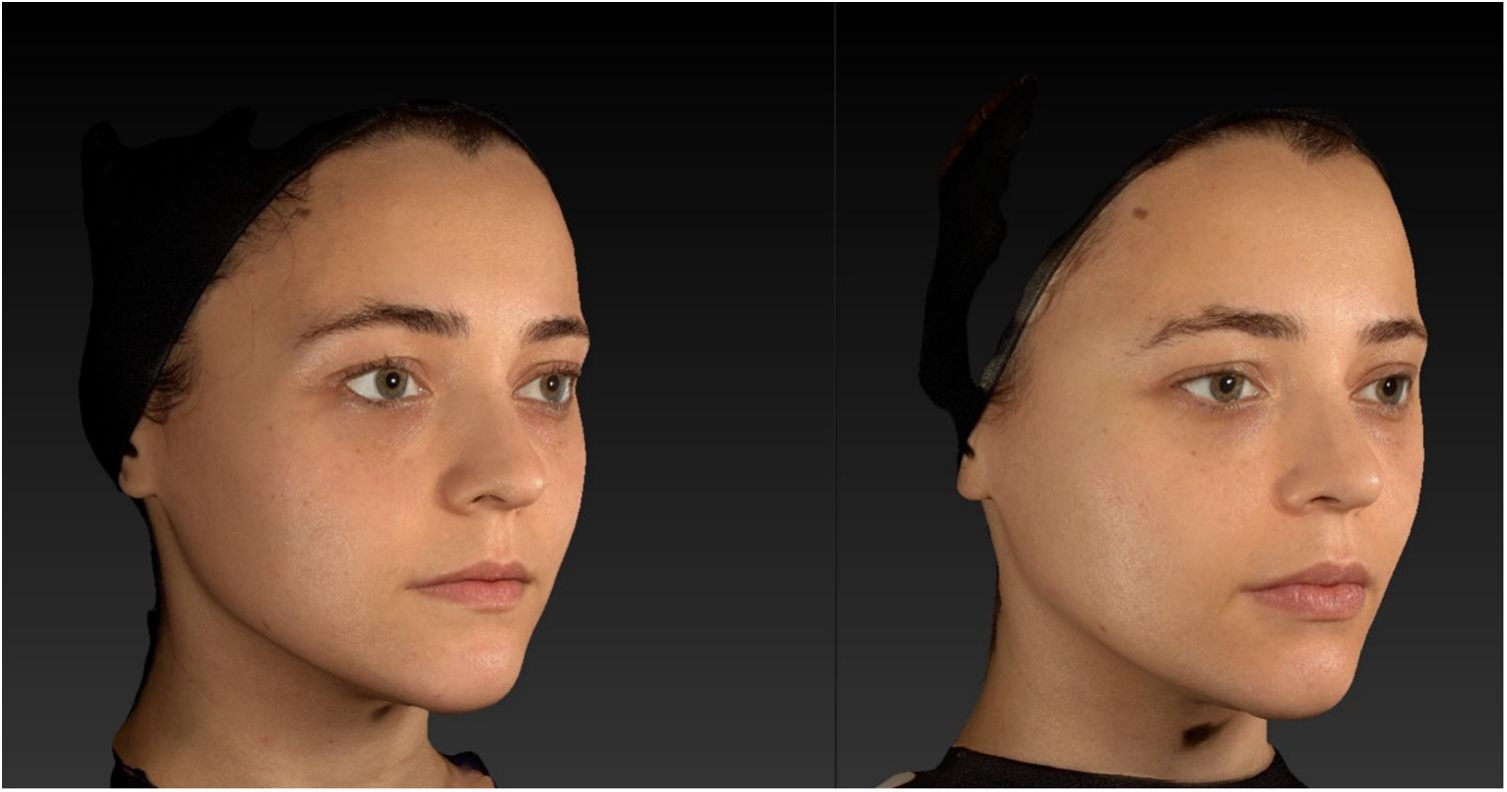
Lateral view showing pre- and post-treatment comparison following interfascial hyaluronic acid (HA) injection in the temporal region. The injection was performed via a 22G × 50 mm blunt-tip cannula inserted at the superior border of the zygomatic arch. Filler was placed within the interfascial plane between the superficial and deep temporal fascia. Post-injection images reveal correction of temporal hollowing, improved contour of the lateral forehead and upper temple, and soft tissue lifting along the lateral brow and upper zygomatic arch. Observable changes include enhanced convexity in the temporal fossa and elevation of the lateral brow tail. Anatomical landmarks include the temporal crest, lateral orbital rim, and zygomatic arch, which serve as reference points for volumetric enhancement.

**Figure 4:**
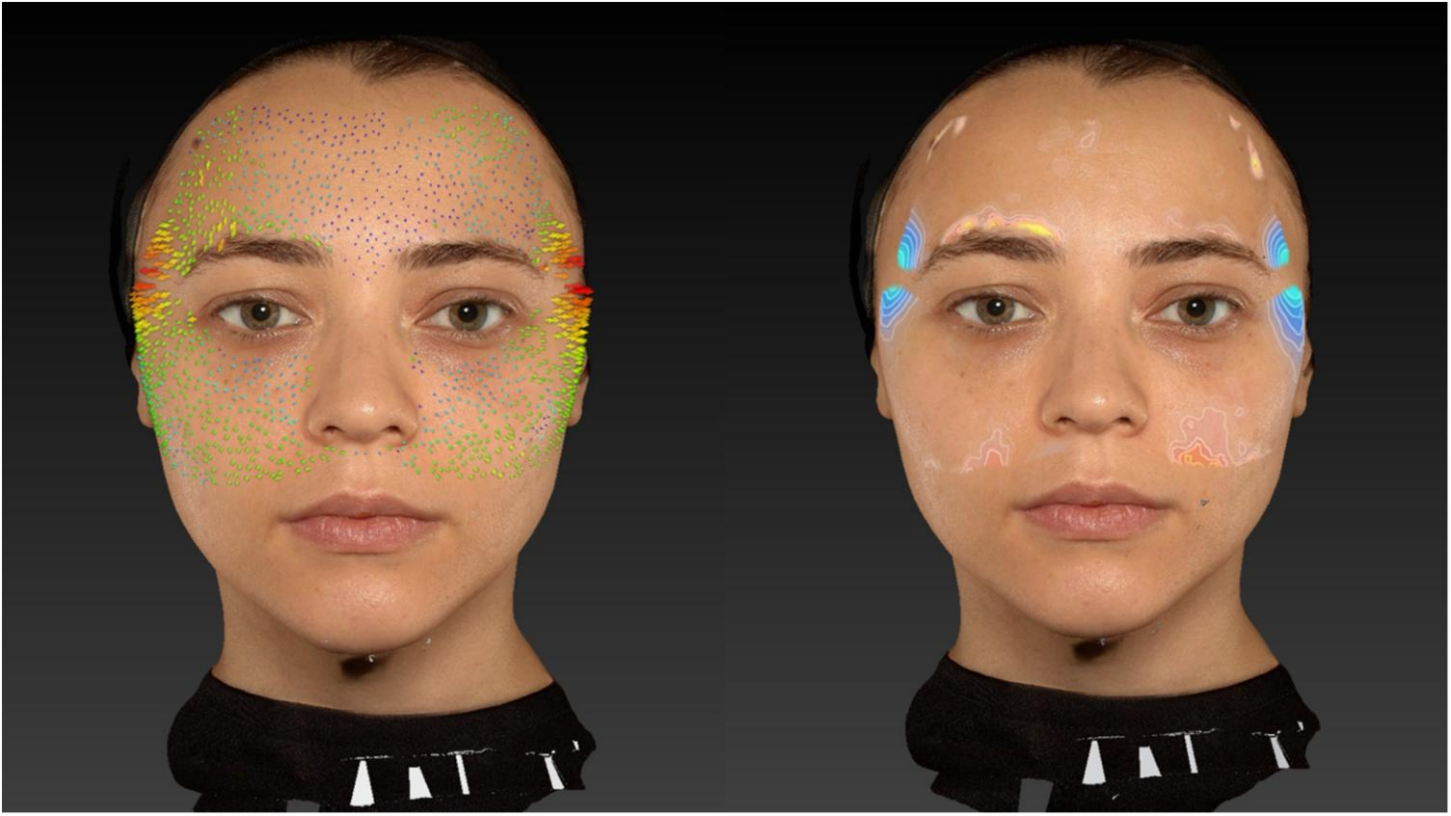
Frontal view before and after interfascial filler placement in the bilateral temporal regions. The image demonstrates restoration of lateral facial fullness, smoothing of temporal depressions, and a lifting effect on the upper third of the face, most notably in the temporal and lateral brow areas. Volumization of the temporal hollowing contributes to a more youthful, balanced facial contour. Key reference points include the supraorbital rim, lateral brow, and anterior temporal line, used to assess post-treatment symmetry and vertical vector lifting. The filler placement was confirmed sonographically within the interfascial compartment.

The results of the study were promising, both in terms of volumization and lifting effects (Figure 3). In all patients, real-time ultrasound confirmed that the filler was deposited within the interfascial layer between the superficial and deep temporal fascia. This was documented in Supplementary Video S1, which demonstrates a representative case of successful cannula guidance and filler distribution within the intended anatomical plane. Vector images demonstrated a clear lifting effect in the upper third of the face, particularly in the temporal region. Volume images further corroborated the effectiveness of this technique in restoring lost volume (Figure 4).

In terms of patient safety, the procedure demonstrated a favorable safety profile. Eighteen patients reported mild pain within 48 h post-injection, while two patients experienced moderate pain. Notably, no patients reported any hematomas or other serious adverse events.

## Discussion

The temporal region is vital to facial rejuvenation as it supports the upper third of the face, but its complex anatomy presents unique challenges for safe and effective aesthetic treatments. Previous literature has identified up to 13 distinct layers in this region, showing intricate anatomical relationships with vascular structures, including the superficial and deep temporal arteries (5). This complexity increases the risk of accidental intravascular injections, vascular occlusion and subsequent ischemic complications, such as vision loss. Traditional injection techniques, whether subcutaneous or supraperiosteal, not only pose these significant risks but also have aesthetic limitations.

Subcutaneous injections may risk inadvertently entering the superficial temporal fascia, which contains the superficial temporal artery, potentially leading to vascular injury. Additionally, in cases of severe temporal hollowing, superficial injections often yield unsatisfactory results, as the filler may be visible under the skin due to insufficient tissue coverage. On the other hand, supraperiosteal injections, while capable of substantial volume restoration, carry a great risk of injuring the deep temporal arteries, and filler displacement due to contractions of the temporalis muscle (6). Moreover, they require a larger volume of product to achieve noticeable surface effects, further complicating the procedure (7, 8).

To overcome these limitations, we propose an interfascial approach that involves injecting the filler between the subcutaneous and supraperiosteal layers. This technique seeks to optimize both safety and efficacy by minimizing the risk of vascular complications and reducing muscle-related displacement. It offers a balanced solution, delivering a noticeable surface effect while ensuring sufficient soft tissue coverage to prevent product visibility. The interfascial layer offers a reproducible and safe environment for filler placement. Notably, recent studies also documented the feasibility and safety of injections between the superficial and deep temporal fasciae: Lee et al. found that filling the interfascial layer is “relatively safe and easy” and Cohn et al. similarly advocate this plane as a safe, efficacious target (9, 10).

An integral aspect of our technique is establishing bone contact at the zygomatic arch to ensure correct placement within the interfascial layer. The anatomical continuity between the periosteum of the zygomatic arch and the deep temporal fascia guides the cannula into the intended anatomical plane, allowing for safe and accurate filler deposition that mimics the natural contours of the face while minimizing the risk of arterial injury or product migration (5, 11).

Additionally, the interfascial plane provides an ideal environment for high-viscoelastic fillers, such as Ultra Volume Rennova®, which are well-suited for tissue volumization and lifting, ensuring that the filler maintains its shape and volume over time without the risk of deformation or displacement. In terms of patient safety, the low incidence of adverse events observed in this study, including minimal reports of pain and no hematomas, highlights the tolerability of the interfascial approach. The use of ultrasound imaging to confirm the injection plane further enhances safety by ensuring precise filler placement. However, we acknowledge that differentiating between the superficial and deep temporal fascia using ultrasound may be challenging in certain cases, particularly due to variations in adipose thickness and acoustic window quality (12, 13).

## Conclusion

The interfascial injection technique described in this study offers a novel and effective approach to facial rejuvenation, particularly in the anatomically and aesthetically challenging temporal region. By leveraging the anatomical relationship between the zygomatic arch and the deep temporal fascia, this technique provides a safer, more efficient, and aesthetically superior alternative to traditional subcutaneous and supraperiosteal injections. The results of this study demonstrate the efficacy of this approach in achieving both lifting and volumization, while maintaining a favorable safety profile. Further studies with larger patient populations are warranted to confirm these findings and to explore the long-term effects of this technique in facial rejuvenation.

## Data Availability

The original contributions presented in the study are included in the article/Supplementary Material, further inquiries can be directed to the corresponding author.

## Supplementary material

Supplementary Video S1 The video illustrates the live advancement of the cannula through the interfascial plane between the superficial and deep temporal fascia, followed by hyaluronic acid deposition. The visualized anechoic filler layer between two hyperechoic fascial boundaries confirms precise interfascial entry and distribution.
